# The Effect of Ankle Foot Orthosis' Design and Degree of Dorsiflexion on Achilles Tendon Biomechanics—Tendon Displacement, Lower Leg Muscle Activation, and Plantar Pressure During Walking

**DOI:** 10.3389/fspor.2020.00016

**Published:** 2020-03-17

**Authors:** Åsa Fröberg, Mattias Mårtensson, Anton Arndt

**Affiliations:** ^1^Division of Orthopaedics and Biotechnology, Department of Clinical Sciences, Intervention and Technology (Clintec), Karolinska Institute, Stockholm, Sweden; ^2^KTH, Biomedical Engineering and Health Systems, Stockholm, Sweden; ^3^The Swedish School of Sport and Health Sciences (GIH), Stockholm, Sweden

**Keywords:** Achilles tendon, ankle foot orthoses, brace, speckle tracking, deformation, EMG, plantar pressure

## Abstract

**Background:** Following an Achilles tendon rupture, ankle foot orthoses (AFO) of different designs are used to protect the healing tendon. They are generally designed to protect against re-rupture by preventing undesired dorsiflexion and to prevent elongation by achieving plantarflexion in the ankle. There is limited knowledge of the biomechanical effects of different AFO designs and ankle angles on the tendon and lower leg muscles.

**Hypothesis:** The hypothesis was that non-uniform displacement in the Achilles tendon, lower leg muscle activity, and plantar pressure distribution would be affected differently in different designs of AFO and by varying the degree of dorsiflexion limitation.

**Study Design:** Controlled laboratory study.

**Methods:** Ultrasound of the Achilles tendon, EMG of the lower leg muscles and plantar pressure distribution were recorded in 16 healthy subjects during walking on a treadmill unbraced and wearing three designs of AFO. Ultrasound speckle tracking was used to estimate motion within the tendon. The tested AFO designs were a rigid AFO and a dorsal brace used together with wedges and an AFO with an adjustable ankle angle restricting dorsiflexion to various degrees.

**Results:** There were no significant differences in non-uniform tendon displacement or muscle activity between the different designs of AFO. For the rigid AFO and the adjustable AFO there was a significant reduction in non-uniform displacement within the tendon and soleus muscle activity as restriction in dorsiflexion increased.

**Conclusion:** The degree of dorsiflexion allowed within an AFO had greater effects on Achilles tendon displacement patterns and muscle activity in the calf than differences in AFO design. AFO settings that allowed ankle dorsiflexion to neutral resulted in displacement patterns in the Achilles tendon and muscle activity in the lower leg which were close to those observed during unbraced walking.

## Introduction

In previous studies of Achilles tendon rupture, short leg casts and ankle foot orthoses (AFO) were used during rehabilitation to protect the healing tendon (Mortensen et al., 1999; Maffulli et al., 2003; Kangas et al., 2007; Nilsson-Helander et al., 2010; Willits et al., 2010; Silbernagel et al., 2012; Schepull and Aspenberg, 2013). In these studies casts and different designs of AFO were used in various combinations, which makes it difficult to get a clear idea about whether some AFO designs are more suitable than others. Different types of AFO used in clinical practice are generally designed to protect against re-rupture by preventing undesired dorsiflexion and to prevent elongation by achieving plantarflexion (Kearney et al., 2011). One design has a rigid outer shell which restricts both dorsiflexion and plantarflexion and the desired ankle angle is achieved by adding heel wedges (Willits et al., 2010; Olsson et al., 2013). Another design has an adjustable foot plate which can be set at different angles allowing a limited range of motion (Nilsson-Helander et al., 2010; Twaddle and Poon, 2013). Thirdly, dorsal braces are used to restrict undesired dorsiflexion while plantarflexion is allowed (Maffulli et al., 2003; Kangas et al., 2007). Previous studies have shown differences in muscle activity in the gastrocnemius and the soleus during walking with a rigid AFO design (Akizuki et al., 2001; Kadel et al., 2004) compared to an adjustable AFO design (Fröberg et al., 2009). Ankle moments as calculated from plantar pressure measurements have been shown to decrease when walking in a rigid AFO (Sandberg et al., 2015), whereas force in the Achilles tendon has been shown to increase when using an adjustable AFO with increasing restriction of dorsiflexion (Fröberg et al., 2009). In these studies no imaging of the Achilles tendon was used.

The Achilles tendon transmits forces from the medial and lateral gastrocnemius and soleus which can be individually activated (Bojsen-Moller and Magnusson, 2015). Ultrasound speckle tracking studies of the Achilles tendon have shown that displacement within the Achilles tendon is non-uniform during walking and that non-uniformity increases with increasing walking speed (Franz et al., 2015). The non-uniform displacement pattern observed in the Achilles tendon is thought to reflect gliding between tendon fascicles (Haraldsson et al., 2008; Arndt et al., 2012; Slane and Thelen, 2014; Franz et al., 2015) and it has been shown to be disturbed in previously ruptured tendons (Fröberg et al., 2017). Fascicle gliding is thought to be important in optimizing force transmission during motion, as the level of gastrocnemius and soleus activity and the angles of the knee and ankle varies (Arndt et al., 2012; Bojsen-Moller and Magnusson, 2015).

The aim was to investigate how non-uniform displacement patterns within the Achilles tendon, muscle activity in the lower leg, and plantar pressure distribution in healthy subjects is affected by the use of three different designs of AFO and by allowing varying degrees of dorsiflexion during walking. The hypothesis was that non-uniform displacement in the Achilles tendon, lower leg muscle activity, and plantar pressure distribution would be affected differently in different designs of AFO and by varying the degree of dorsiflexion limitation. It was hypothesized that non-uniform displacement would be (1) lower in the AFO conditions compared to unbraced walking and (2) lower in AFO settings restricting dorsiflexion.

## Methods

**Figure 1:**
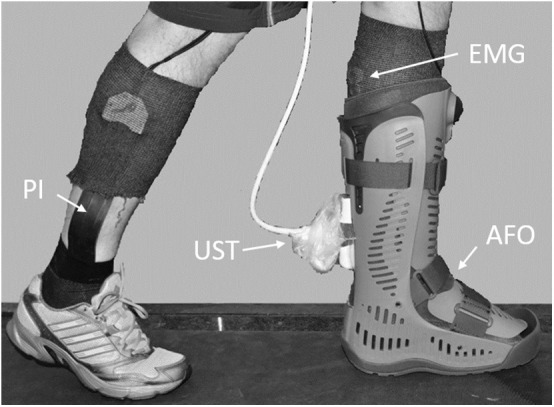
Experimental set up. EMG, emg electrodes; UST, ultrasound transducer; PI, pressure insole; AFO, rigid ankle foot orthosis.

Ultrasound of the Achilles tendon, EMG of the lower leg muscles and plantar pressure distribution were recorded in 16 healthy subjects during walking on a treadmill without an AFO and wearing three designs of AFO (Figure 1). Subjects were eight males (mean ± standard deviation (SD) age: 45 ± 3 years, height: 183 ± 7 cm, body mass: 82 ± 13 kg) and eight females (mean ± SD age: 44 ± 3 years, height: 170 ± 5 cm, body mass: 66 ± 10 kg). The Regional Ethics Committee approved the study (2016/1970-31) and subjects gave written informed consent.

### AFO Conditions

**Figure 2:**
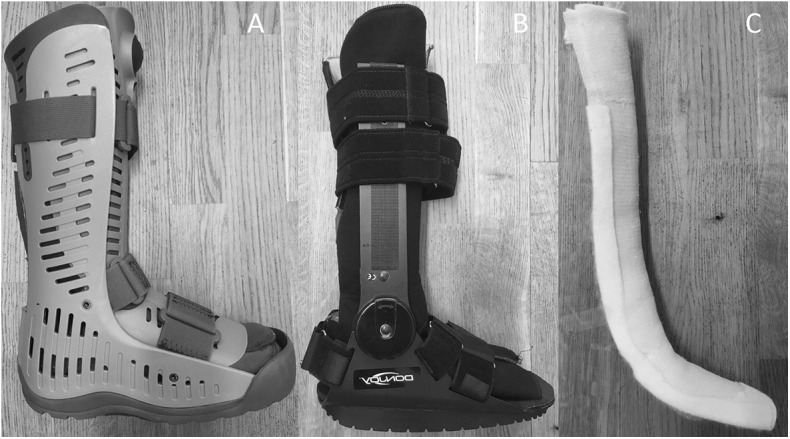
The three different designs of ankle foot orthoses that were tested. **(A)** rigid AFO, **(B)** adjustable AFO, and **(C)** dorsal brace.

Three different designs of AFO were tested during walking on a treadmill at 2 km/h (Figure 2). The first design was an AFO with a rigid outer shell and a rocker bottom sole (Rebound Air Walker, Össur, Reykjavik, Iceland) which restricted both dorsiflexion and plantarflexion. It was tested using zero, two or three 10 mm heel wedges. The second design was an AFO with an adjustable rocker bottom foot plate (ROM Walker, DJO Global, Vista, USA) which was tested using three different settings with dorsiflexion limited to 10° dorsiflexion, 10° plantarflexion or 30° plantarflexion respectively, while plantarflexion was unrestricted. Thirdly, a dorsal brace was made for each subject using 10 layers of casting tape (Scotchcast Plus, 3M Health Care, St Paul, USA) with the ankle in neutral position. The brace was fixed to the leg using cohesive bandage (Mollelast haft, Lohmann & Rauscher International GmbH & Co, Rengsdorf, Germany) and thus restricted dorsiflexion to neutral. It was used together with regular running shoes and was tested with no heel wedge and with a 10 mm wedge in the shoe. As the brace was worn inside the shoes, there was a soft resistance to plantarflexion. All subjects also walked and ran in stocking feet (unbraced) at 2 and 10 km/h, respectively. A slow walking speed (2 km/h) was chosen to facilitate a relaxed walking pattern at all AFO conditions. The test order was randomized. The right foot was tested in all subjects. For the AFO conditions subjects wore a running shoe with a 10 mm wedge on the opposite foot to compensate for leg length difference. After change of condition, subjects were allowed to get accustomed to the new AFO before recordings started. The AFO were adjusted so that the ultrasound probe could be placed on the Achilles tendon. For the rigid AFO a 17 × 5.5 cm opening was cut in the plastic shell over the Achilles tendon, but the compressive air bladders were left intact. For the adjustable AFO, an opening was cut in the soft material covering the Achilles tendon and the distal Velcro strap was moved a few cm proximally.

### Synchronization and Electromyography

In order to synchronize EMG data collection, plantar pressure measurement and ultrasound acquisition, a data collection configuration was created using Spike2 software (7.09a ×86, Cambridge Electronic Design, Cambridge, UK). At start of data collection the data acquisition unit (Micro 1401, Cambridge Electronic Design) generated a square wave signal that was fed into the ultrasound machine and a trigger signal that was sent to the plantar pressure measurement software (25.3.6, Pedar-x online, Novel GmbH, Munich, Germany). EMG activity was measured in the medial and lateral gastrocnemius, the soleus and in the tibialis anterior using adhesive bipolar surface electrodes (BlueSensorN, Ambu, Ballerup, Denmark) with an inter-electrode distance of 20 mm. The electrodes and cables were further fixed using compressive stockings before the AFO were fitted (Figure 1). Data was collected at 3,000 Hz and sent by wireless transmission to a receiver box (TeleMyo 2400R G, Noraxon, Scottsdale, USA), then converted to digital in the data acquisition unit and saved on a PC. Three 15 s recordings were made for each walking condition. EMG data was exported as text files and imported into MATLAB (R2014a, MathWorks Inc., Natick, USA). EMG raw data and corresponding ultrasound images were visually inspected and ten strides with good quality EMG and ultrasound were chosen for each walking condition and subject, respectively. A stride was defined as heelstrike to heelstrike and the time for heelstrike for the chosen strides were identified in the plantar pressure data. EMG raw data was moved to zero, rectified, smoothed using 200 point adjacent averaging and normalized to mean peak EMG of unbraced running at 10 km/h. Peak EMG values were identified on the resulting curves and mean and SD of the peak values were calculated. Mean EMG curves for all strides and all subjects were calculated for each muscle and walking condition.

### Plantar Pressure

Each subject was fitted with a pair of pressure measurement insoles (Pedar-xf-16/R system, Novel GmbH) of appropriate size which were held in place by compressive stockings inside the AFO. For the unbraced conditions an extra pair of compressive stockings was worn. Between each change in walking condition the insoles were calibrated so that the unloaded foot resembled zero pressure. Three 15 s recordings at a sampling frequency of 100 Hz were made for each walking condition. Data were saved and then exported as asc-files into MATLAB for further analysis. The time for heelstrikes for the right foot were identified from the plantar pressure data. For further analysis of plantar pressure the foot was divided into three regions according to recommendations (Barnett, 1998), where the forefoot equaled the distal 40% of the total length, midfoot equaled the middle 30% and rearfoot equaled the proximal 30%. Forefoot and rearfoot pressure were then calculated as a mean of the pressure sensors in each region, respectively. To allow for comparisons between subjects, pressure data was normalized and expressed as a percentage of mean peak pressure during unbraced walking at 2 km/h. Peak forefoot and rearfoot pressures for the ten strides previously chosen for each subject and walking condition were identified and averaged. Strides with data artifacts were removed. Mean forefoot pressure curves were calculated for all subjects and walking conditions and then used to identify toe off time for each walking condition.

### Ultrasound

Ultrasound speckle tracking is a method developed for measuring deformation in tissue. Speckle tracking algorithms use the speckle pattern present in all ultrasound images to track deformation between frames in ultrasound sequences (Arndt et al., 2012; Slane and Thelen, 2014; Franz et al., 2015; Fröberg et al., 2017). For ultrasound imaging, a 9L linear array transducer (GE Healthcare, Horten, Norway) connected to a Vivid-q ultrasound machine (GE Healthcare) was fixed over the Achilles tendon using a fixation device that supported the probe to prevent it from swaying. The transducer was placed so that the posterior process of the distal tibia was visible in the distal end of all images and it remained fixed in the same position for all walking conditions. For each walking condition, three 15 s B-mode ultrasound acquisitions (10 MHz, 40 FPS, depth 3 cm) were made using a standoff pad. The time of the synchronization signal visible in the ultrasound image was noted for each recording using EchoPAC (110.1.2, GE Healthcare). Motion files were converted to HDF format and imported into MATLAB. Two dimensional motion estimation was performed with a MATLAB speckle tracking algorithm which has previously been validated (Fröberg et al., 2017), using a kernel size of 52λ (laterally) ×25λ (axially), 80% kernel overlap and normalizes cross correlation as similarity measure. For each subject the same 10 strides that were previously chosen for EMG and plantar pressure evaluation were analyzed. A 25 mm region of interest (ROI) was placed in the middle of the visible tendon portion and adjusted so that it covered the full thickness of the tendon. Average displacement within the superficial and deep thirds of the ROI was computed. All resulting displacement curves were interpolated to obtain data sets of equal length and then mean curves for superficial and deep displacement for all subjects were computed using MATLAB. Peak displacement during stance phase was defined as displacement during the time from the beginning of dorsiflexion to maximum dorsiflexion as determined from the displacement curves. Minimum and maximum displacement values were identified on all superficial and deep displacement curves for all evaluated strides, and peak displacement was calculated as the difference between the minimum and maximum values. Differential displacement was calculated as the difference in deep and superficial peak displacement. Mean and SD of peak values were calculated for all subjects.

### Statistical Analysis

A two-tailed paired *t*-test was performed for pairwise comparison of displacement in the superficial and deep layers of the tendon for all walking conditions using SPSS (Statistics 24, IBM, Armonk, USA). A repeated measures analysis of variance was performed in SPSS to establish if there were any differences in superficial displacement, deep displacement, differential displacement, EMG activity, forefoot pressure, and rearfoot pressure between walking conditions. Two comparisons were made between the different designs of AFO and unbraced walking. Firstly the conditions with least restriction in dorsiflexion (rigid AFO 0 wedges, adjustable AFO 10° dorsiflexion and dorsal brace 0 wedges) were compared amongst themselves and with unbraced walking. Secondly the conditions restricting dorsiflexion most (rigid AFO 3 wedges and adjustable AFO 30° plantarflexion) were compared amongst themselves and with unbraced walking. The dorsal brace was not included in the second comparison as it did not place the ankle in the same degree of plantarflexion. To test the influence of dorsiflexion range of motion within each AFO design, the different settings within each AFO were compared to each other and to unbraced walking. Mauchly's test of Sphericity was used to test the assumption of sphericity. When the assumption of sphericity was violated the Greenhouse-Geisser correction was used. The Bonferroni correction was used to make pairwise comparisons between condition means for superficial displacement, deep displacement, differential displacement, and EMG activity. Forefoot pressure and rearfoot pressure for all AFO conditions were normalized against mean pressure during unbraced walking, which was set to 100%. Mean forefoot and rearfoot pressure and 95% confidence intervals (CI) were then calculated for all AFO conditions and compared to unbraced walking. If the CI did not include 100%, the difference was considered to be significant.

## Results

**Figure 3:**
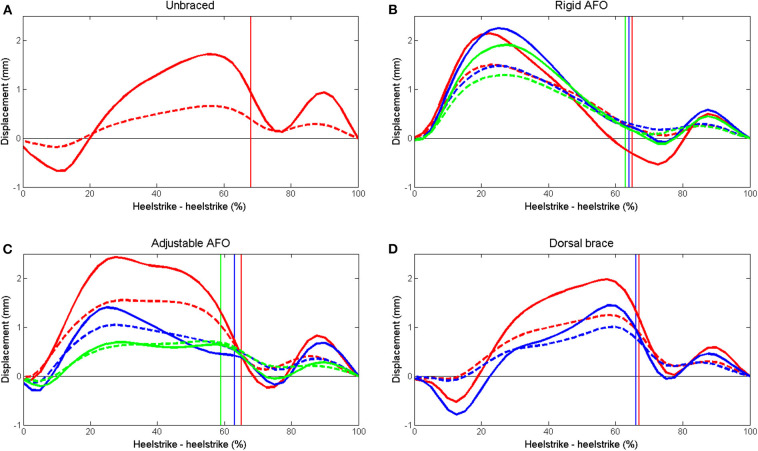
Mean displacement in the superficial (dashed) and deep (solid) layers of the Achilles tendon during walking. **(A)** unbraced walking, **(B)** rigid AFO with 0 wedges (red), 2 wedges (blue) and 3 wedges (green), **(C)** adjustable AFO with ankle dorsiflexion limited to 10° dorsiflexion (red), 10° plantarflexion (blue) and 30° plantarflexion (green), and **(D)** dorsal brace with 0 wedges (red) and 1 wedge (blue). Toe off time is indicated for each setting by a vertical line in the corresponding color.

**Figure 4:**
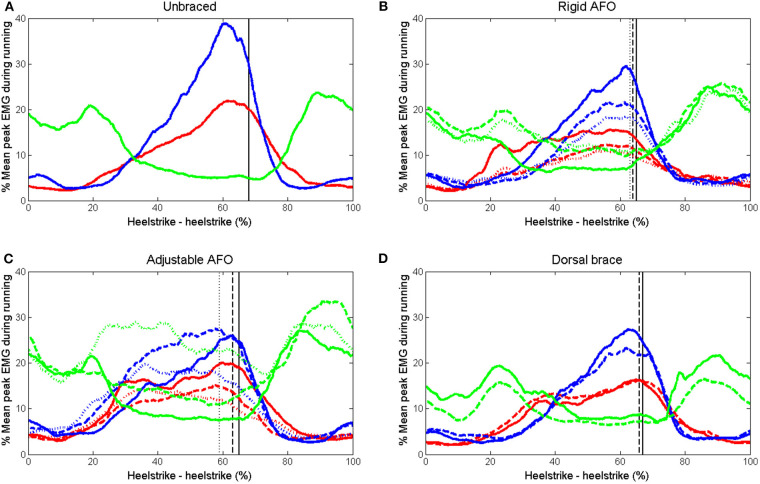
Mean EMG curves for medial gastrocnemius (blue), soleus (red), and tibialis anterior (green) during walking. **(A)** unbraced walking **(B)** rigid AFO with 0 wedges (solid), 2 wedges (dashed), and 3 wedges (dotted). **(C)** adjustable AFO with ankle dorsiflexion limited to 10° dorsiflexion (solid), 10° plantarflexion (dashed), and 30° plantarflexion (dotted) **(D)** dorsal brace with 0 wedges (solid) and 1 wedge (dashed). Toe off time is indicated for each setting by a vertical line with the corresponding line format.

**Figure 5:**
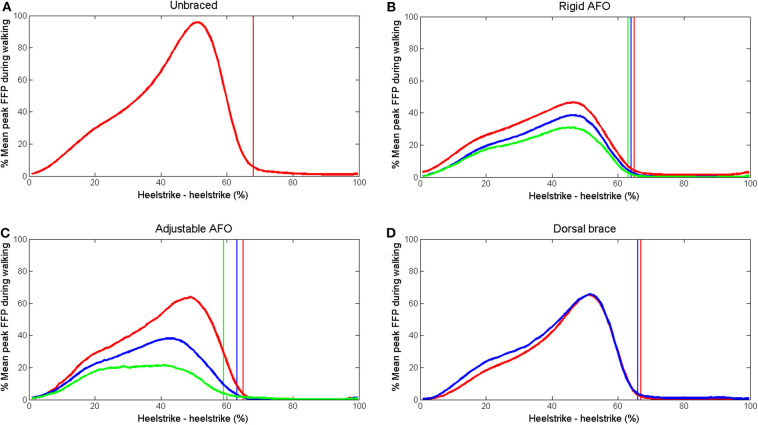
Mean forefoot pressure during walking. **(A)** unbraced walking **(B)** rigid AFO with 0 wedges (red), 2 wedges (blue) and 3 wedges (green). **(C)** adjustable AFO with ankle dorsiflexion limited to 10° dorsiflexion (red), 10° plantarflexion (blue), and 30° plantarflexion (green) **(D)** dorsal brace with 0 wedges (red) and 1 wedge (blue). Toe off time is indicated for each setting by a vertical line in the corresponding color.

Mean curves for superficial and deep displacement in the Achilles tendon, EMG for soleus, medial gastrocnemius and tibialis anterior and forefoot pressure for all subjects are shown in Figures 3–5. Mean and SD of peak displacement in the Achilles tendon, peak EMG activity of the lower leg muscles and peak plantar pressure are shown in Table 1. Mean peak displacement in the deep parts of the tendon was significantly larger than superficial displacement for all walking conditions (*p* < 0.001). There were no significant differences in differential displacement or muscle activity between the rigid AFO 0 wedges, the adjustable AFO 10°dorsiflexion or the dorsal brace 0 wedges or between the rigid AFO 3 wedges and the adjustable AFO 30° plantarflexion.

**Table 1:** Mean and SD of peak displacement in the Achilles tendon, peak EMG activity in the soleus, medial, and lateral gastrocnemius and tibialis anterior and peak plantar pressure.

**Condition**	Superficial disp^*^	Deep disp^*^	**Δ disp**	**EMG med gast**	**EMG lat gast**	**EMG sol**	**EMG tib ant**	**Forefoot P**		**Rearfoot P**	
	**Mean ± SD (mm)**	**Mean ± SD (mm)**	**Mean ± SD (mm)**	**Mean ± SD (%)**	**Mean ± SD (%)**	**Mean ± SD (%)**	**Mean ± SD (%)**	**Mean ± SD (%)**	**95% CI**	**Mean ± SD (%)**	**95% CI**
Unbraced	1.1 ± 0.4 *rAFO 0,2 & 3 wdg,* *aAFO 10df & 10pf,* *db 0 & 1wdg*	3.2 ± 0.8 *aAFO 30 pf*	2.2 ± 0.7 *aAFO 30pf*	58 ± 24 *rAFO 0,2 & 3wdg,* *aAFO 10pf & 30pf, db*	27 ± 12 *rAFO 0,2 & 3wdg*	31 ± 7 *rAFO 2 & 3wdg,* *aAFO 30pf, db 0wdg*	38 ± 18 *aAFO 10pf, aAFO 30pf*	100	–	100	–
rAFO 0 wdg	1.9 ± 0.7 *ub*	3.7 ± 0.7	1.7 ± 0.7 *rAFO 3wdg*	43 ± 18 *ub, rAFO 2wdg, rAFO 3wdg*	18 ± 11 *ub, rAFO 3wdg*	29 ± 13 *rAFO 2wdg, rAFO 3wdg*	41 ± 24	48 ± 17 *rAFO 2wdg, rAFO 3wdg*	39–58	76 ± 15 *rAFO 2wdg, rAFO 3wdg*	68–84
rAFO 2 wdg	1.7 ± 0.6 *ub*	3.3 ± 0.9	1.5 ± 0.8	34 ± 14 *ub, rAFO 0wdg*	14 ± 9 *ub*	19 ± 5 *ub, rAFO 0wdg*	43 ± 23	42 ± 19 *rAFO 0 wdg, rAFO 3wdg*	31–52	99 ± 21 *rAFO 0wgd*	88–110
rAFO 3 wdg	1.7 ± 0.6 *ub*	3.1 ± 0.7	1.4 ± 0.7 *rAFO 0wdg*	31 ± 14 *ub, rAFO 0wdg*	14 ± 8 *ub, rAFO 0wdg*	18 ± 7 *ub, rAFO 0wdg*	45 ± 28	34 ± 18 *rAFO 0wdg, rAFO 2wdg*	24–43	103 ± 21 *rAFO 0wgd*	92–114
aAFO 10°df	2.1 ± 0.7 *ub, aAFO 10pf & 30pf*	4.1 ± 0.6 *aAFO 10pf, aAFO 30pf*	2.0 ± 0.7 *aAFO 30pf*	41 ± 16 *ub*	23 ± 13	31 ± 15	46 ± 27	69 ± 22 *aAFO 10pf, aAFO 30pf*	57–82	73 ± 15	65–82
aAFO 10°pf	1.6 ± 0.7 *ub,aAFO 10df*	3.1 ± 0.8 *aAFO 10df, aAFO 30pf*	1.6 ± 0.7	47 ± 17 *aAFO 30pf*	26 ± 16	24 ± 9	52 ± 28 *ub*	45 ± 21 *aAFO 10df, aAFO 30pf*	33–57	82 ± 26	68–96
aAFO 30°pf	1.2 ± 0.5 *aAFO 10df*	2.2 ± 0.5 *ub, aAFO 10df, aAFO 10pf*	1.1 ± 0.5 *ub,aAFO 10df*	31 ± 16 *ub, aAFO 10pf*	23 ± 13	23 ± 10 *ub*	55 ± 28 *ub*	27 ± 18 *aAFO 10df, aAFO 10pf*	17–36	85 ± 27	70–100
DB 0 wdg	1.7 ± 0.7 *ub*	3.7 ± 0.8	2.0 ± 0.6	42 ± 20 *ub*	21 ± 16	24 ± 10 *ub*	44 ± 45	65 ± 23 *DB 1wdg*	53–77	77 ± 23 *DB 1wdg*	65–89
DB 1wdg	1.5 ± 0.6 *ub*	3.4 ± 0.8	1.9 ± 0.5	37 ± 17 *ub*	19 ± 14	24 ± 12	32 ± 16	72 ± 26 *DB 0wdg*	58–86	88 ± 30 *DB 0wdg*	72–104

*EMG is normalized to mean peak EMG during unbraced running at 10 km/h. Statistical differences between conditions are shown in italics (p < 0.05). disp, displacement; Δ disp, differential displacement; P, pressure, normalized to pressure during unbraced walking at 2 km/h. ub, unbraced; rAFO, rigid AFO; aAFO, adjustable AFO; db, dorsal brace; wdg, wedges; df, dorsiflexion; pf, plantarflexion*.

* *Mean peak displacement in the deep parts of the tendon was significantly larger than in superficial parts for all walking conditions (p < 0.001)*.

### Rigid AFO

Differential displacement was significantly reduced (*p* = 0.03) from condition rigid AFO 0 wedges to rigid AFO 3 wedges. EMG activity was significantly reduced in the medial (*p* = 0.006) and lateral (*p* = 0.042) gastrocnemius and soleus (*p* = 0.006) from condition rigid AFO 0 wedges to rigid AFO 3 wedges. Forefoot pressure showed a progressive reduction with each extra wedge (*p* < 0.014).

### Adjustable AFO

Differential displacement was significantly reduced as range of motion decreased from adjustable AFO set at 10° dorsiflexion to 30° plantarflexion (*p* < 0.001). EMG activity in the soleus showed a trend to decrease as dorsiflexion limitation increased and there was a significant reduction from condition unbraced walking to adjustable AFO setting 30° plantarflexion (*p* = 0.024). Medial gastrocnemius activity varied between settings. Tibialis anterior activity significantly increased from condition unbraced walking to adjustable AFO set at 10° plantarflexion (*p* = 0.03) or 30° plantarflexion (*p* = 0.008).

### Dorsal Brace

During walking with the dorsal brace with or without wedge, differential displacement did not differ significantly compared to unbraced walking. Medial gastrocnemius and soleus EMG activity significantly decreased when the dorsal brace was used compared to unbraced walking (*p* < 0.027).

## Discussion

A non-uniform displacement pattern with greater displacement in deep compared to superficial parts of the Achilles tendon was present for all walking conditions (Figure 3). When the rigid and the adjustable AFO were used, tendon displacement became more uniform, EMG activity in the soleus decreased and forefoot pressure decreased as the ankle was placed in gradually increasing plantarflexion and less dorsiflexion was permitted (Table 1). No significant differences in differential displacement or lower leg muscle activity were found between the different designs of AFO for either of the compared conditions (Table 1).

Peak superficial Achilles tendon displacement was significantly larger for all AFO conditions compared to unbraced walking, except for the adjustable walker set to 30° plantarflexion (Table 1). The same tendency was observed for deep displacement although this was not significant. As the AFO are designed to limit ankle range of motion, this was unexpected. It was subjectively observed that participants altered their walking patterns between the different walking conditions, but no motion analysis was performed to verify this. For the rigid and the adjustable AFO, displacement appeared to increase more rapidly from ~5–25% of stride with an earlier peak displacement compared to unbraced walking and the dorsal brace condition (Figure 3). The initial negative displacement seen in the unbraced and dorsal brace conditions (Figure 3) did not occur in these AFO which may indicate that no initial plantarflexion occured directly after heelstrike. The rigid and the adjustable AFO both have a rocker bottom sole which may affect this phase of stance. Such gait modifications are at present not fully understood and their effect upon tendon displacement remains to be investigated.

Despite differences in their design, no differences in the non-uniform displacement pattern of the Achilles tendon were found between the AFO designs for either of the compared conditions (Table 1). AFO are thought to protect the Achilles tendon by reducing passive tension by restricting dorsiflexion and by reducing active loading by decreasing muscle activity (Akizuki et al., 2001; Kearney et al., 2011). For a rigid AFO, ankle plantarflexion torque during walking estimated from EMG has previously been shown to decrease with the addition of heel wedges (Akizuki et al., 2001). In the adjustable AFO dorsiflexion was limited by setting the foot plate in different ankle angles and when it was set in plantarflexion there was no support under the heel. For the most plantarflexed positions it was difficult to achieve a relaxed walking pattern and participants needed to support their weight entirely on the forefoot. Force in the Achilles tendon during walking has previously been shown to increase compared to unbraced walking in the most plantarflexed postions for this AFO design (Fröberg et al., 2009). Therefore, some difference in tendon displacement patterns was expected between these AFO designs. Instead the degree of dorsiflexion limitation within each AFO design seemed to affect tendon displacement patterns more. For the rigid and the adjustable AFO differential displacement was significantly reduced as limitation in dorsiflexion increased (Table 1). The dorsal brace was tested with or without a single 10 mm wedge and the difference between the two conditions was perhaps too small for this effect to be seen.

There were no significant differences in gastrocnemius, soleus, or tibialis anterior activity between the different AFO designs, but differences were observed between different degrees of dorsiflexion limitation. EMG activity in the medial and lateral gastrocnemius and soleus was reduced compared to unbraced walking when the rigid AFO was used, and the reduction was more pronounced as dorsiflexion was limited with added wedges (Table 1). This finding is similar to previous results shown for this AFO design (Akizuki et al., 2001; Kadel et al., 2004). When the adjustable AFO was used, EMG activity in soleus showed a tendency to decrease as dorsiflexion limitation gradually increased (Table 1). In contrast, gastrocnemius activity was higher when the ankle was placed in 10° plantarflexion than for 10° dorsiflexion (Table 1). This has been shown previously for this AFO design (Fröberg et al., 2009). As mentioned above, when this AFO is set in 30° plantarflexion participants had to support on their toes and bend their knees to avoid limping. Muscle activity in the soleus and the gastrocnemius during isometric plantarflexion has previously been shown to vary depending on the knee angle (Arndt et al., 1998). Differences in muscle activity in the soleus and the gastrocnemius between the different AFO designs might be explained by differences in the walking patterns. Muscle activity in the tibialis anterior increased compared to unbraced walking when the adjustable AFO was set to 10° plantarflexion or beyond. This may have been due to the AFO design with the plantarflexed foot plate and an increased effort to dorsiflex the foot during swing phase to avoid stumbling.

During walking the Achilles tendon produces plantarflexion moment around the ankle and the forefoot is used for push-off against the ground. Therefore the amount of forefoot pressure produced during walking has been suggested as an important parameter to evaluate regarding Achilles tendon loading (Kearney et al., 2011). During barefoot walking, differential displacement has previously been shown to increase with higher walking speeds (Franz et al., 2015) where push-off is expected to be more forceful. Forefoot pressure was reduced compared to unbraced walking for all AFO conditions. For the rigid AFO and the adjustable AFO the reduction in forefoot pressure became more pronounced as the ankle was placed in successively more plantarflexion and less dorsiflexion was allowed in conjunction with reduced non-uniform displacement. This result is similar to the results reported in a previous study, where forefoot pressure was also shown to correlate to the degree of dorsiflexion allowed within an AFO (Kearney et al., 2011).

In rat studies of Achilles tendon ruptures it has been shown that, weight bearing on the injured limb and exercise on a treadmill resulted in earlier formation of mature repair tissue with thicker and longitudinally organized collagen (Bring et al., 2007) and tendons with higher peak force and stiffness (Andersson et al., 2009; Eliasson et al., 2012), than if the limbs were unloaded or immobilized in a cast. Continuous activity without immobilization also seemed to be more effective than immobilization and intermittent training in stimulating healing (Andersson et al., 2009). It is not known how these findings translate into humans, but it indicates that loading which mimics unbraced walking may be a good stimulus for healing. The AFO settings with least restriction of dorsiflexion resulted in non-uniform tendon deformation patterns that most resembled those during unbraced walking. Neuromuscular electrical stimulation or exercise has been shown to partly counteract reduction in muscle mass and strength which result from limb immobilization (Alkner and Tesch, 2004; Dirks et al., 2018). Further it has been demonstrated in animal models that calf muscles undergo more atrophy if they are immobilized in shortened positions compared to lengthened positions (Sjostrom et al., 1979; Rantanen et al., 1999). AFO designs and settings which permitted more dorsiflexion resulted in less reduction in muscle activity and less calf muscle shortening which may be beneficial in preventing muscle atrophy.

Following an Achilles tendon rupture it has been common practice to place the ankle in plantarflexion in a cast or AFO to adapt the tendon ends and protect the healing tendon from elongation (Nilsson-Helander et al., 2010; Willits et al., 2010; Schepull et al., 2012; Olsson et al., 2013). There are a few studies of surgical treatment of Achilles tendon ruptures where dorsiflexion to neutral was allowed early. A dorsal brace limiting dorsiflexion to neutral applied either the day after surgery or after 2 weeks and with full weight bearing after 2–3 weeks was compared to cast immobilization and no increase in re-ruptures (Kangas et al., 2003; Maffulli et al., 2003) or tendon elongation (Kangas et al., 2003) were found. The adjustable AFO design set to limit dorsiflexion to neutral 2 weeks postoperatively was compared to cast treatment and no difference in re-rupture rate or tendon elongation were found (Mortensen et al., 1999). These studies suggest that mobilizing the ankle to neutral within 2 weeks following surgical treatment of an Achilles tendon rupture is safe, while evidence regarding non-surgical treatment is lacking.

There are limitations to this study. Validation of Speckle tracking on tendon tissue has previously been done *in vitro* on porcine tendon samples (Chernak and Thelen, 2012; Fröberg et al., 2017) and *in vivo* validation is lacking. It has been reported that speckle tracking has a tendency to systematically underestimate displacement in tendon tissue (Chernak and Thelen, 2012; Fröberg et al., 2017). Peak superficial and deep displacement during stance phase were defined as displacement during the time from the beginning of dorsiflexion to maximum dorsiflexion as determined from the displacement curves. Differential displacement was calculated as the difference between peak superficial and deep displacement. Although this potentially introduces a risk of error due to a time shift between superficial and deep displacement, no major time shifts were observed.

Pressure in-sole systems are limited to measuring force vectors that are perpendicular to the sensors and therefore there is a risk for underestimation of forces if the insoles are placed on sloping surfaces (Spooner et al., 2010). In this study AFO conditions with different plantar surface slopes were compared and therefore there might be measurement errors present. Tendon deformation patterns were observed in healthy participants who were not limited by pain or caution and were able to fully load their braced leg. This may not be the case following an Achilles tendon rupture. It was observed that participants changed walking patterns with the use of the different types of AFO, but as motion analysis was not used this could not be analyzed further. The rigid AFO had to be adjusted to fit the ultrasound probe over the Achilles tendon. The opening may have affected the biomechanical properties of the AFO, but no apparent instabilities were observed.

In conclusion, the degree of dorsiflexion allowed within an AFO had greater effects on Achilles tendon displacement patterns and muscle activity in the calf than differences in AFO design. AFO settings that allowed ankle dorsiflexion to neutral resulted in displacement patterns in the Achilles tendon and muscle activity in the lower leg which were close to those observed during unbraced walking. Further research is needed to establish if these effects are related to clinical benefits.

## Data Availability Statement

The datasets generated for this study are available on request to the corresponding author.

## Ethics Statement

The studies involving human participants were reviewed and approved by the Stockholm Regional Ethics Committe (2016/1970-31). The patients/participants provided their written informed consent to participate in this study.

## Author Contributions

ÅF contributed to conception, design and data collection of the study, performed data analysis, and wrote the manuscript. MM contributed to conception, design and data collection of the study, performed data analysis, and assisted in writing the manuscript. AA contributed to conception, design and data collection of the study, assisted in data analysis, and assisted in writing the manuscript.

### Conflict of Interest

The authors declare that the research was conducted in the absence of any commercial or financial relationships that could be construed as a potential conflict of interest.
